# Metabolic Master Switch: Pyruvate Carboxylase Fuels Antimicrobial Resistance and Virulence in Foodborne *Staphylococcus aureus*

**DOI:** 10.3390/foods14152566

**Published:** 2025-07-22

**Authors:** Zifeng Mai, Jiahui Li, Zeqiang Zhan, Xiaorong Tian, Wanwan Hou, Mu He, Chunlei Shi

**Affiliations:** State Key Laboratory of Microbial Metabolism, Department of Food Science & Technology, School of Agriculture & Biology, Shanghai Jiao Tong University, Shanghai 200240, China; maizifeng@sjtu.edu.cn (Z.M.); jhli1997@sjtu.edu.cn (J.L.); zhanzeqiang@sjtu.edu.cn (Z.Z.); tianxr@sjtu.edu.cn (X.T.); houwanwan@sjtu.edu.cn (W.H.); 018150210012@alumni.sjtu.edu.cn (M.H.)

**Keywords:** *Staphylococcus aureus*, pyruvate carboxylase, antimicrobial resistance, biofilm, TCA cycle, purine metabolism

## Abstract

*Staphylococcus aureus*, a major cause of foodborne illness globally, presents significant challenges due to its multidrug resistance and biofilm-forming capabilities. Pyruvate carboxylase (PycA), a metabolic master switch linking glycolysis and the tricarboxylic acid (TCA) cycle, is a potential target for controlling *S. aureus*. In this study, a *pycA* mutant was constructed and analyzed using phenotypic assays and proteomics to investigate its role in virulence and antimicrobial resistance. The results showed that deletion of *pycA* in the foodborne methicillin-resistant strain ATCC BAA1717 resulted in a 4- to 1024-fold reduction in resistance to β-lactams, aminoglycosides, and macrolides; a 23.24% impairment in biofilm formation; and a 22.32% decrease in staphyloxanthin production, a key antioxidant essential for survival in oxidative food environments. Proteomic analysis revealed downregulation of the TCA cycle, purine biosynthesis, surface adhesins (FnbA/B, SasG), and β-lactamase (BlaZ), linking PycA-mediated metabolism to phenotypes relevant to food safety. These findings underscore the importance of PycA as a metabolic regulator crucial for *S. aureus* resilience in food systems, suggesting novel strategies to combat foodborne staphylococcal infections through metabolic interference.

## 1. Introduction

*Staphylococcus aureus* is a prevalent foodborne pathogen responsible for staphylococcal food poisoning through the production of heat-stable enterotoxins [1]. In addition to its toxigenic potential, the pathogenicity of *S. aureus* is mediated by an arsenal of virulence factors, including surface adhesins, the antioxidant pigment staphyloxanthin (STX), and various immune evasion molecules, which facilitate effective colonization, biofilm formation, and persistence in hostile environments [2]. Worsening the situation is the widespread emergence of methicillin-resistant *S. aureus* (MRSA) in food environments [3,4], which exhibits multidrug resistance and severely limits therapeutic options [5]. Given its dual threat of toxicity and resistance, *S. aureus* presents a significant challenge to food safety and public health, highlighting the urgent need for novel antimicrobial strategies targeting both resistance mechanisms and virulence regulation.

Recent studies have highlighted the critical role of central metabolic pathways in regulating both antimicrobial resistance and virulence. For example, glycolysis mitigates energy depletion during antimicrobial stress, contributing to resistance against cyclic lipopeptide antibiotics such as polymyxin B and daptomycin [6]. Moreover, glycolysis enables *S. aureus* to sustain energy metabolism and redox balance under nitric oxide-induced hypoxic conditions, thereby facilitating immune evasion and promoting virulence during infection [7]. Similarly, disruption of the TCA cycle leads to ATP depletion and reduced membrane potential, promoting the formation of persister cells and enhancing tolerance to multiple antimicrobial classes [8,9,10].

Pyruvate carboxylase (PycA) is a key enzyme in central carbon metabolism, catalyzing the carboxylation of pyruvate to oxaloacetate, thereby linking glycolysis to the TCA cycle. Previous genome-wide screening studies have identified *pycA* as a critical gene involved in virulence attenuation in *S. aureus* [11]. In a murine systemic infection model, disruption of *pycA* markedly reduced bacterial burden in vivo and mitigated infection-induced tissue necrosis. Moreover, the *pycA* mutant displayed increased susceptibility to macrophage-mediated clearance [12]. During a preliminary screen for virulence-associated genes using a transposon mutant library in *S. aureus*, we unexpectedly found that mutation of the *pycA* gene led to a noticeable decrease in both virulence and antimicrobial resistance. However, its role in regulating antimicrobial resistance remains unclear. Here, we investigated the impact of *pycA* deletion on antimicrobial resistance, virulence, and metabolic pathways in *S. aureus*, providing insights into its potential as a control target for food safety management.

## 2. Materials and Methods

### 2.1. Bacterial Strains and Plasmids

The strains and plasmids used in this study are listed in Table 1. All strains were stored at –80 °C in tryptic soy broth (TSB) supplemented with 25% glycerol. Unless otherwise specified, strains were cultured in TSB at 37 °C prior to use. For the *pycA*-complemented (c*pycA*) and *pycA*-overexpression strains (WT-p*pycA*), *pycA* expression was induced with 0.15 μg/mL anhydrotetracycline.

### 2.2. Mutated Strain Construction

In this study, deletion of the *pycA* in *S. aureus* BAA1717 (wild-type, WT) was performed using a modified λ-Red homologous recombination strategy, as previously reported with minor modifications [17]. Briefly, upstream and downstream homologous arms of the *pycA* were amplified by PCR and ligated into the *Sal* I and *Kpn* I sites of the temperature-sensitive plasmid pKZ2. The resulting recombinant plasmid was first introduced into *E. coli* JTU006 for restriction-modification and subsequently electroporated into *S. aureus* BAA1717. Homologous recombination facilitated the deletion of the *pycA* locus, and the Δ*pycA* mutant was verified by PCR. For gene complementation and overexpression assays, the full-length *pycA* gene along with its native promoter was cloned into the shuttle vector pCL55 to construct the recombinant plasmid pCL55-*pycA*. This plasmid was subsequently introduced into both the Δ*pycA* mutant and the WT for functional complementation and overexpression, respectively.

### 2.3. Minimum Inhibitory Concentration Determination

According to the Clinical and Laboratory Standards Institute (CLSI) guidelines [18], the minimum inhibitory concentrations (MICs) of antimicrobials against *S. aureus* strains were determined using the broth microdilution method. In brief, antimicrobials were two-fold serially diluted in TSB across 96-well microtiter plates. Bacterial suspensions were prepared from overnight cultures and diluted to a final concentration of 1 × 10^6^ CFU/mL. Equal volumes of the antimicrobial dilutions and bacterial suspensions were added to each well. The plates were incubated at 37 °C for 24 h. The MIC was defined as the lowest antimicrobial concentration that visibly inhibited bacterial growth.

### 2.4. Time-Kill Curve Assay

Time-kill assays were performed on WT and Δ*pycA* strains following previously described protocols [19]. Antimicrobials were used at 1/4 MIC, based on the MIC values determined for the WT BAA1717 strain.

### 2.5. Biofilm Quantification

Biofilm formation was assessed using crystal violet staining in a 96-well microtiter plate [20]. Briefly, bacterial suspensions were diluted in TSB to achieve a final concentration of 1 × 10^6^ CFU/mL, and 200 μL of this mixture was added to each well, followed by incubation at 37 °C for 24 h. After incubation, the wells were gently washed three times with phosphate-buffered saline (PBS, pH 7.4) to remove planktonic cells, then fixed at 60 °C for 15 min. The biofilms were stained with 0.1% crystal violet for 15 min, washed with PBS, and decolorized with 200 μL of absolute ethanol for 5 min. Absorbance was measured at 595 nm using a microplate reader. TSB without bacterial inoculation served as the blank control. All experiments were performed in triplicate, with five technical replicates per group.

### 2.6. Staphyloxanthin Quantification

Staphyloxanthin (STX) quantification was performed using a previously described method with minor modifications [21]. *S. aureus* strains were inoculated into brain heart infusion (BHI) medium and incubated at 37 °C with shaking at 200 rpm for 30 h. After incubation, 2 mL of the culture was centrifuged to collect the bacterial pellet, which was subsequently washed three times with PBS (pH 7.4). The pellet was resuspended in PBS, and the bacterial suspension was adjusted to a final concentration of 10^9^ CFU/mL. The normalized bacterial pellet was resuspended in 800 μL of 99% methanol and mixed thoroughly. The mixture was incubated at 55 °C in the dark for 2 h, followed by centrifugation. A 200 μL aliquot of the supernatant was collected, and the absorbance at 462 nm was measured using a microplate reader to quantify STX, as this wavelength corresponds to its characteristic absorbance peak. All experiments were conducted in triplicate.

### 2.7. 2,2-Diphenyl-1-Picrylhydrazyl Free Radical Scavenging Activity Assay

The 2,2-diphenyl-1-picrylhydrazyl (DPPH) radical scavenging activity was evaluated using a modified version of a previously described method [22]. *S. aureus* was cultured overnight in TSB, and the bacterial cells were subsequently harvested and washed three times with PBS (pH 7.4). The cell suspension was then standardized to ensure equal bacterial counts across all experimental groups. Cell disruption was performed by sonication on ice (200 W, 3 s on/10 s off, 60 cycles). The lysates were then centrifuged, and the supernatants were collected for antioxidant analysis. For the DPPH assay, 30 μL of each sample was mixed with 170 μL of 0.1 mM DPPH in ethanol in a 96-well plate. PBS served as the blank control. The reaction mixtures were incubated in the dark at room temperature for 30 min, and absorbance was measured at 490 nm using a microplate reader. All experiments were conducted in triplicate.

### 2.8. L-Aspartate Auxotrophy Assay

The minimum essential medium (MEM) for *S. aureus* growth was prepared following previously described protocols [23]. *S. aureus* strains were cultured in 96-well plates containing either MEM alone or MEM supplemented with 100 μg/mL L-aspartate. The plates were incubated at 37 °C under static conditions for 24 h. Bacterial growth was measured by optical density (OD) at 600 nm using a microplate reader. The experiment was conducted with three independent biological replicates. The composition of MEM is provided in Table S1.

### 2.9. Growth Curve Measurement

An overnight culture of *S. aureus* was harvested and diluted in fresh TSB to a final bacterial concentration of 1 × 10^6^ CFU/mL. Bacterial growth was monitored at 37 °C for 26 h using a growth curve analyzer, with OD_600_ readings recorded every 30 min. Each condition was measured in five technical replicates, and the experiment was repeated in three independent biological replicates.

### 2.10. Real-Time PCR Analysis

Total RNA was extracted from 12 h *S. aureus* cultures using the SPARKeasy Bacteria RNA Kit (Sparkjade Biotechnology, Jinan, China). Complementary DNA (cDNA) was synthesized with the HiScript^®^ III RT SuperMix for qPCR (+gDNA wiper) kit (Vazyme Biotech, Nanjing, China), according to the manufacturer’s instructions. Quantitative PCR (qPCR) and reverse transcription qPCR (RT-qPCR) were performed to evaluate the relative expression of genes using 2 × SYBR Green qPCR Mix (with ROX) (Sparkjade Biotechnology, Jinan, China). Gene expression levels were normalized to 16S rRNA as the internal control, and foldchanges were calculated using the 2^−ΔΔCt^ method. All experiments were conducted with three independent biological replicates. Primer sequences are listed in Table S2.

### 2.11. Scanning Electron Microscopy Analysis

Scanning electron microscopy (SEM) was conducted according to a previous protocol with slight modifications [24]. *S. aureus* was cultured in TSB at 37 °C for 12 h. Bacterial cells were washed with PBS (pH 7.4) and fixed in 2.5% (*v*/*v*) glutaraldehyde at 4 °C for 8–12 h. The samples were then dehydrated through a graded ethanol series (30%, 50%, 70%, 90%, and 100%, *v*/*v*), with each step lasting 10 min; the 30% and 100% steps were repeated twice. The dehydrated samples were stored in a desiccator and sputter-coated with gold for SEM analysis.

### 2.12. Transmission Electron Microscopy Analysis

Transmission electron microscopy (TEM) analysis was conducted according to a previous protocol [25]. *S. aureus* cultures were fixed in 2.5% (*v*/*v*) glutaraldehyde at 4 °C for 8–12 h, followed by three washes with PBS (pH 7.4). Post-fixation was carried out using 1% osmium tetroxide for 1 h at room temperature. The samples were then dehydrated through a graded ethanol series (30%, 50%, 70%, 90%, and 100%, *v*/*v*), with each step lasting 10 min. Dehydrated cells were embedded in epoxy resin, and ultrathin sections (70–90 nm) were prepared using an ultramicrotome. These sections were stained with uranyl acetate and lead citrate before being examined under a transmission electron microscope.

### 2.13. Intracellular ATP Analysis

Intracellular ATP levels were measured using an ATP assay kit (Beyotime, Beijing, China) with minor modifications. *S. aureus* strains were standardized to a final concentration of 1 × 10^9^ CFU/mL and washed three times with PBS (pH 7.4). Cells from 2 mL of culture were collected by centrifugation, resuspended in 400 μL lysis buffer with several 2 mm steel beads, and vortexed on ice for 15 min. After centrifugation, 60 μL of the supernatant was used for ATP detection following the kit instructions. ATP concentration was calculated using a standard curve.

### 2.14. Proteomic Analysis

Total proteins from the WT and Δ*pycA* strains were extracted, quantified using the bicinchoninic acid (BCA) assay, and digested with trypsin. The resulting peptides were desalted, dried, and reconstituted in a solvent containing indexed retention time (iRT) standard peptides for LC-MS analysis. Approximately 500 ng of peptides per sample were separated using a Vanquish Neo Nano-scale Ultra Performance Liquid Chromatography (nano-UPLC) system coupled with an Astral mass spectrometer (Thermo Fisher Scientific, Waltham, MA, USA) and analyzed in the data-independent acquisition (DIA) mode. The MS data were processed using the DIA-NN software against the *S. aureus* UniProt database, with quantification based on a 2 m/z isolation window and a 1% false discovery rate (FDR) threshold. Differentially expressed proteins (|log_2_FC| ≥ 0.5, *p* < 0.05) were identified and further analyzed using Gene Ontology (GO), the Kyoto Encyclopedia of Genes and Genomes (KEGG), principal component analysis (PCA), and statistical methods.

### 2.15. A549 Cells Infection Assay

A549 is a human lung adenocarcinoma epithelial cell line that is widely used in cytotoxicity testing and drug screening assays. The A549 cell infection assay was conducted following a previously established protocol, with slight modifications [26]. Briefly, A549 cells were seeded into 96-well plates at a density of 2 × 10^4^ cells per well and incubated for 16 h. The culture medium was then replaced with fresh Dulbecco’s Modified Eagle Medium (DMEM) supplemented with 10% fetal bovine serum (FBS). Subsequently, *S. aureus* strains were added at a multiplicity of infection (MOI) of 100, and the cells were co-incubated for 6 h. Cell viability was assessed using calcein AM/PI dual staining (Beyotime, Beijing, China), which allowed for the differentiation of live and dead cells via fluorescence microscopy. Untreated cells served as the negative control. Cytotoxicity was evaluated by measuring lactate dehydrogenase (LDH) release after 6 h of incubation using a commercial LDH assay kit (Beyotime, Beijing, China). Supernatants were collected for analysis. The A549 cell line was purchased from Wuhan Procell Life Science & Technology Co., Ltd. (Wuhan, China).

### 2.16. Statistical Analyses

Statistical analyses were conducted using GraphPad Prism 10.1.2 (GraphPad Software, San Diego, CA, USA). Group differences were evaluated using unpaired t-tests or one-way ANOVA, as appropriate. Results with *p*-values less than 0.05 were deemed statistically significant.

## 3. Results

### 3.1. Deletion of pycA Increases Antimicrobial Susceptibility in S. aureus

Deletion of *pycA* significantly increased the susceptibility of *S. aureus* to a broad range of antimicrobials (Table 2). Notably, the MICs of Δ*pycA* for β-lactams, including oxacillin, ampicillin, and amoxicillin, were reduced by 64- to over 1024-fold compared to the WT. Enhanced sensitivity was also observed to aminoglycosides, including kanamycin sulfate and amikacin (4- to 64-fold reductions), as well as for macrolides, with erythromycin and azithromycin MICs decreased by 128-fold. Complementation with *pycA* (c*pycA*) partially restored antimicrobial resistance to WT levels, while overexpression of *pycA* (WT-p*pycA*) further elevated MICs, indicating enhanced resistance. These results indicate that *pycA* may indirectly contribute to *S. aureus* resistance to β-lactams, aminoglycosides, and macrolides. This observation is further corroborated by time-kill assays (Figure S1), which reveal that all tested antimicrobials exert enhanced bactericidal activity against the Δ*pycA* relative to the WT.

Antimicrobial resistance in *S. aureus* is typically multifactorial. Previous studies have shown that downregulation of central carbon metabolism in *S. aureus* leads to ATP depletion, promoting the formation of persister cells and enhancing antimicrobial resistance [10,27]. However, *pycA*, encoding a key enzyme bridging glycolysis and the TCA cycle, appears to play an inverse role. Deletion of *pycA* reversed resistance to multiple antimicrobial classes in the WT, suggesting that PycA may act upstream to modulate the expression of established resistance determinants. Notably, the partial restoration of resistance upon c*pycA* indicates that *pycA* deletion may involve potentially irreversible metabolic reprogramming, thereby hindering full restoration of the original metabolic state and resistance phenotype. Furthermore, the increased sensitivity to β-lactams observed in the Δ*pycA* mutant may be attributed to reduced *blaZ* expression rather than *mecA*, as no difference in cefoxitin resistance was detected between Δ*pycA* and WT. In the case of aminoglycosides, the heightened susceptibility observed in Δ*pycA* may be linked to decreased activity of 3’,5’’-aminoglycoside phosphotransferase (type III), an enzyme that inactivates aminoglycosides via phosphorylation [28]. Similarly, the increased susceptibility to macrolide antimicrobials may be explained by impaired expression of macrolide phosphotransferases or reduced activity of ATP-dependent efflux pumps [29]. Given the observed ATP reduction in the Δ*pycA* mutant, it is plausible that energy deficiency compromises efflux efficiency, leading to intracellular accumulation of macrolides and enhanced bactericidal effects. Collectively, these findings indicate that PycA acts as a key regulator of antimicrobial resistance in *S. aureus*, potentially via metabolic alterations.

### 3.2. PycA Is Dispensable for Growth Under Nutrient-Rich Conditions but Essential in the Absence of L-Aspartate

**Figure 1:**
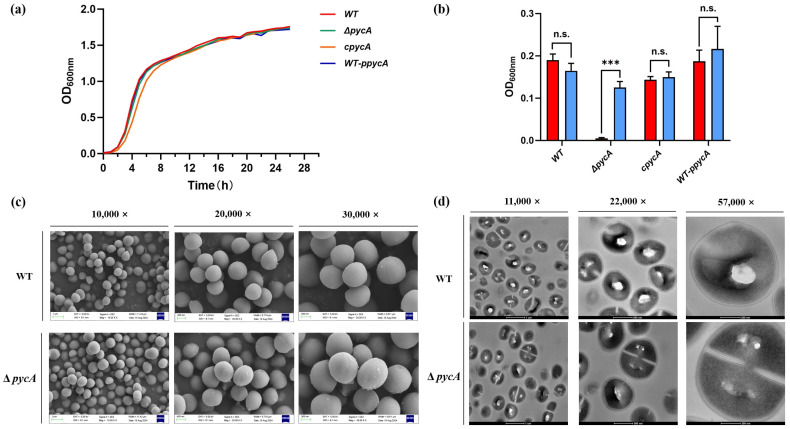
The effect of PycA on the growth of *S. aureus*. (**a**) Growth curves of *S. aureus* strains in TSB medium; (**b**) L-ASP auxotrophy assay in MEM with or without 100 μg/mL L-ASP supplementation; (**c**) SEM images showing surface morphology at 10,000×, 20,000×, and 30,000× magnification; (**d**) TEM images of cellular ultrastructure at 11,000×, 22,000×, and 57,000× magnification. *** *p* < 0.001; n.s., not significant.

To rule out the possibility that the reduced antimicrobial resistance in the Δ*pycA* strain was due to a growth defect, growth curves were evaluated in TSB. As shown in Figure 1a, deletion of *pycA* does not significantly impair bacterial growth under standard culture conditions, as further supported by the absence of significant morphological changes observed by SEM and TEM (Figure 1c,d). PycA catalyzes the carboxylation of pyruvate to form oxaloacetate, a precursor for aspartic acid (ASP) synthesis via aspartate aminotransferase [30]. To determine whether *pycA* deletion disrupts ASP biosynthesis, a nutritional limitation assay was conducted. As shown in Figure 1b, Δ*pycA* exhibited significantly reduced growth without exogenous L-ASP, while supplementation restored growth to levels comparable to the c*pycA* strain. No growth defects were observed in the WT or WT-p*pycA* strains, suggesting that *pycA* is essential for survival under ASP-limited conditions.

These findings indicate that *pycA* deletion does not affect *S. aureus* growth under nutrient-rich conditions but impairs the biosynthesis of ASP. Under normal physiological conditions, the concentration of free ASP is approximately 2.8 μg/mL in human serum [31] and around 2 μg/mL in mouse serum [32], concentrations that are insufficient to support the growth of the Δ*pycA*. ASP is an essential metabolic intermediate, and the pathogen must rely on endogenous ASP synthesis to sustain purine biosynthesis and survival [33]. Therefore, PycA is essential for the survival and virulence of *S. aureus* during infection and represents a potential target for novel antimicrobial strategies.

### 3.3. Impaired Virulence isConfirmed in the ΔpycA Mutant

#### 3.3.1. Deletion of *pycA* Inhibits Biofilm Formation by *S. aureus* In Vitro

**Figure 2:**
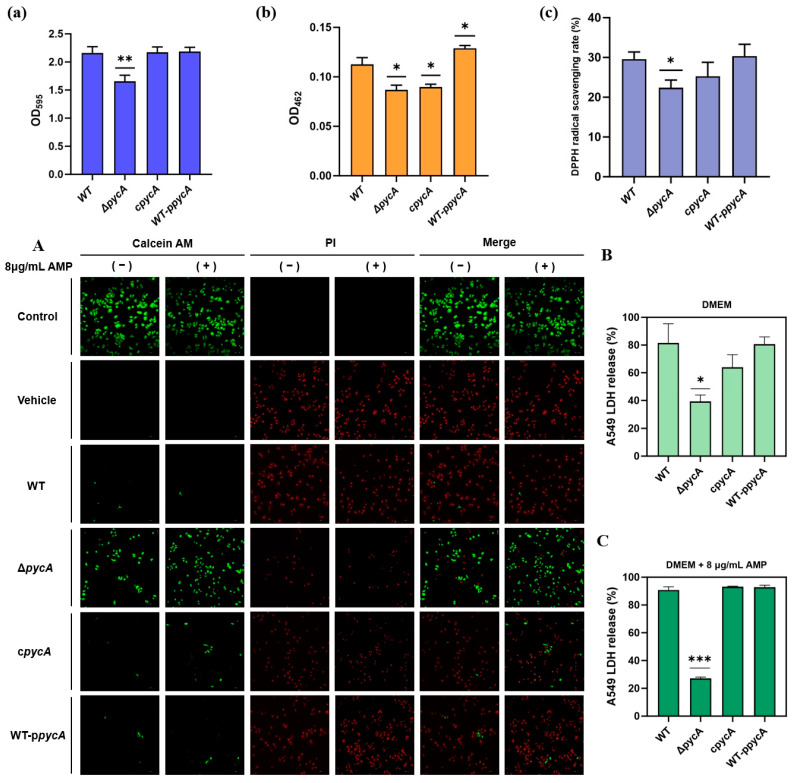
In vitro assessment of virulence-associated phenotypes in *S. aureus* strains. (**a**) Quantification of biofilm formation; (**b**) quantification of STX production; (**c**) total antioxidant capacity assessed using DPPH radical scavenging assay; (**A**) fluorescence microscopy of A549 cells after infection with *S. aureus* strains, in the absence (−) or presence (+) of 8 μg/mL ampicillin; and (**B**,**C**) LDH release assays measuring cytotoxicity of different *S. aureus* strains to A549 cells under (**B**) normal and (**C**) ampicillin-treated conditions. * *p* < 0.05; ** *p* < 0.01; *** *p* < 0.001 compared to the WT group.

In addition to mediating resistance phenotypes, PycA may also influence key virulence-associated factors that facilitate surface adhesion and persistence, which are critical for *S. aureus* survival and contamination in food processing environments. Among these, biofilm formation is a well-recognized virulence strategy in *S. aureus* [34,35]. Quantitative analysis revealed that *pycA* deletion significantly reduced biofilm biomass by 23.24% compared to the WT (Figure 2a). This defect was fully restored in the c*pycA* strain, indicating that PycA plays a critical role in maintaining biofilm-forming capacity. Given that the biofilm matrix impedes antimicrobial penetration and supports persister cell survival [36], the diminished biofilm formation in the Δ*pycA* strain likely contributes to its increased antimicrobial susceptibility and attenuated pathogenicity.

#### 3.3.2. Deletion of *pycA* Inhibits STX Production and Reduces Total Antioxidant Capacity in *S. aureus*

During cultivation, visual inspection indicated a reduction in STX production in the Δ*pycA* compared to the WT. Quantitative analysis further confirmed a 22.32% decrease in STX levels in the Δ*pycA* strain, while overexpression of *pycA* increased STX production by 15.18% (Figure 2b). However, complementation of *pycA* did not fully restore STX levels to those observed in the WT. STX, a golden carotenoid pigment unique to *S. aureus*, enhances resistance to oxidative stress through its antioxidant properties by scavenging reactive oxygen species [37]. Additionally, STX intercalates into the bacterial membrane, reducing fluidity and impairing the insertion of antimicrobial peptides, thus enhancing bacterial resistance [38,39]. To further investigate oxidative stress resistance, the total antioxidant capacity of the strains was measured using the DPPH radical scavenging assay. Consistent with the reduction in STX levels, the Δ*pycA* strain exhibited a 24.26% decrease in total antioxidant activity (Figure 2c), which was partially restored in the c*pycA* strain. These findings further confirm that reduced STX levels in the Δ*pycA* strain impair its antioxidant capacity, which may compromise its resistance to host innate immune defenses and oxidative disinfectants, potentially contributing to its attenuated virulence.

#### 3.3.3. Deletion of *pycA* Reduces the Cytotoxicity of *S. aureus* and Its Survival Under Antimicrobial Pressure

Confocal microscopy and LDH assays demonstrated that the Δ*pycA* exhibited significantly reduced cytotoxicity toward A549 cells compared to the WT, as evidenced by increased live-cell staining and lower LDH release (Figure 2A,B). The complementation of *pycA* restored its cytotoxicity to WT levels. Under ampicillin treatment (8 μg/mL), only the Δ*pycA* failed to induce significant cell death (Figure 2A,C). These findings suggest that *pycA* is required for full virulence in host cells and enhances bacterial survival under antimicrobial stress. The reduced cytotoxicity and antimicrobial resistance observed in the Δ*pycA* strain indicate that PycA plays a critical role as a metabolic node linking central metabolism to both virulence and drug resistance. Previous studies have demonstrated that PycA is essential for the intracellular replication of *Listeria monocytogenes*, as its deletion impairs bacterial growth within mammalian host cells [40]. Similarly, in *S. aureus*, PycA replenishes the TCA cycle and supports the biosynthesis of essential precursors such as amino acids, nucleotides, and cell wall components. Disruption of this metabolic flux likely limits the availability of key intermediates required for the synthesis of virulence factors and other biomolecules necessary for survival within mammalian host cells.

### 3.4. Key Proteins Involved in the Regulation of Virulence and Antimicrobial Resistance via Proteomic Analysis

#### 3.4.1. Deletion of *pycA* Affects the Overall Metabolism of *S. aureus*

**Figure 3:**
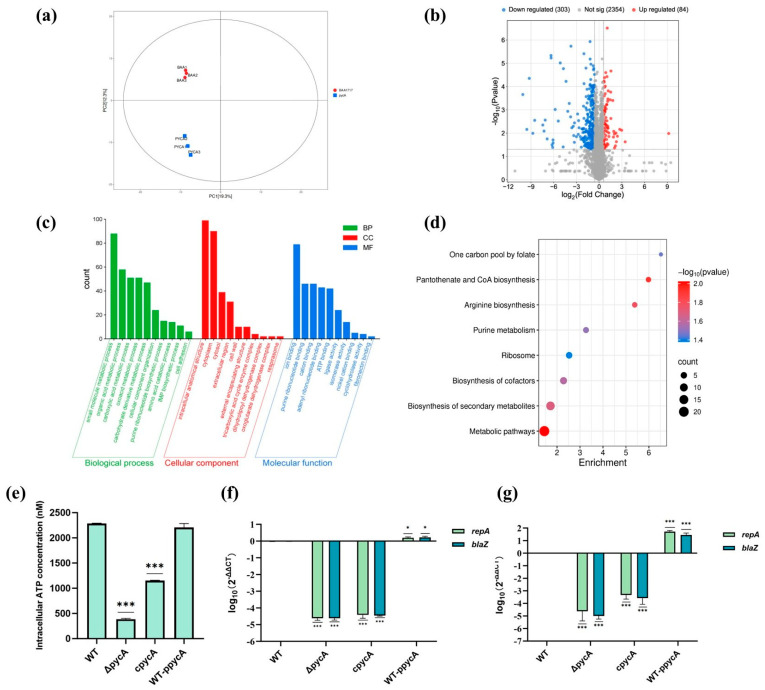
Proteomic and functional analysis of the Δ*pycA* mutant in *S. aureus*. (**a**) PCA showing distinct clustering of WT and Δ*pycA*; (**b**) volcano plot showing differentially expressed proteins between WT and Δ*pycA*; (**c**) GO enrichment analysis of differentially expressed proteins; (**d**) KEGG pathway enrichment analysis of differentially expressed proteins; (**e**) measurement of intracellular ATP levels in *S. aureus* strains; and (**f**,**g**) qPCR (**f**) and RT-qPCR (**g**) analysis of *repA* and *blaZ* genes associated with resistance plasmids. * *p* < 0.05; *** *p* < 0.001 compared to the WT group.

PCA analysis revealed distinct proteomic profiles between WT and Δ*pycA* (Figure 3a). Comparative proteomic analysis identified 2741 unique peptides across the proteomes. Among these, 84 proteins were significantly upregulated and 303 were significantly downregulated in the Δ*pycA* (Figure 3b). GO analysis revealed significant changes in biological processes such as carboxylic acid metabolism, purine ribonucleotide metabolism, amino acid metabolism, and cell adhesion (Figure 3c). Notably, the enrichment of ATP-binding proteins in the Molecular Function category further suggests impaired energy metabolism in the Δ*pycA* strain.

KEGG pathway enrichment analysis revealed that the differentially expressed proteins were predominantly involved in central metabolic pathways, particularly those related to pantothenate and CoA biosynthesis, purine metabolism, and arginine biosynthesis (Figure 3d). These pathways are tightly linked to central carbon metabolism and precursor synthesis, supporting the hypothesis that *pycA* deletion disrupts oxaloacetate production and its downstream metabolic network. Additionally, the enrichment of the ribosomal pathway and folate-mediated one-carbon metabolism indicates potential impairments in protein and nucleotide biosynthesis.

Further analysis of functional modules of differentially expressed proteins (Table 3) revealed that key TCA cycle enzymes (FumC, SucC, SucD, and Mqo) were significantly downregulated in the Δ*pycA*. This suggests that reduced oxaloacetate production suppresses TCA activity. Consistently, intracellular ATP quantification showed a dramatic reduction in the Δ*pycA* compared to the WT, confirming the energy-deficient phenotype associated with *pycA* deletion (Figure 3e). Notably, despite this significant decline in ATP levels, the growth of *S. aureus* remained unaffected. A plausible explanation is that under energy-limiting conditions, the bacterium may prioritize survival by downregulating non-essential ATP-consuming processes, such as virulence factor production, thereby conserving energy for essential cellular functions [41]. Similar findings have been reported in other studies, where inactivation of the TCA cycle in *S. aureus* does not impair its growth under nutrient-rich conditions, supporting that *pycA* deletion-induced ATP reduction does not affect growth rate [10]. While TCA cycle inactivation has been linked to persister formation via ATP depletion [9], the Δ*pycA* strain exhibited increased antimicrobial susceptibility, indicating that the metabolic changes induced by *pycA* deletion may be insufficient to induce a classical persister-like state.

Enzymes involved in de novo purine biosynthesis were also significantly downregulated in Δ*pycA*. This may be attributed to reduced ASP synthesis, as ASP is an essential amino donor in the purine nucleotide biosynthetic pathway [42]. Furthermore, purine biosynthesis is a highly energy-consuming process, requiring six ATP molecules per IMP produced [43]. ATP limitation may further restrict this pathway in Δ*pycA*. Purine metabolism is central to both the virulence and antimicrobial resistance in *S. aureus*. It is upregulated during biofilm formation to support energy generation and extracellular DNA synthesis, both critical for biofilm stability [44]. Moreover, *purF* and *purN* contribute to ATP and ppGpp production, which regulate the formation of antimicrobial-tolerant persister cells and activate virulence-related regulators such as the SaeRS [45,46]. These findings suggest that PycA may promote *S. aureus* virulence by sustaining purine biosynthesis, thereby linking central carbon metabolism to both energy metabolism and virulence gene regulation.

#### 3.4.2. Deletion of *pycA* Downregulates Plasmid-Associated Antimicrobial Resistance Proteins

In the Δ*pycA*, the expression levels of β-lactam resistance-associated proteins (BlaZ, BlaI, and BlaR1) were significantly decreased (Table 3). In *S. aureus* BAA1717 (the WT strain), the *blaZ* is encoded on the plasmid pUSA300HOUMR [47], which also carries resistance genes against macrolides and aminoglycosides (Figure S2). To examine whether *pycA* influences plasmid maintenance, qPCR and RT-qPCR were used to assess the expression of the plasmid replication initiator gene *repA* and the resistance gene *blaZ* (Figure 3f,g). Both *repA* and *blaZ* expression were significantly reduced in the Δ*pycA* strain and markedly upregulated upon *pycA* overexpression. However, complementation of *pycA* did not fully restore their expression to WT levels, suggesting that PycA exerts a threshold-dependent or partially irreversible effect on plasmid stability. The coordinated downregulation of *repA* and *blaZ* following *pycA* deletion indicates that metabolic impairment, likely due to reduced oxaloacetate availability, may disrupt nucleotide pool balance and cellular energy status. As resistance plasmids impose a metabolic burden on *S. aureus* by diverting resources from essential cellular functions [48], the metabolic stress caused by *pycA* deletion may reduce plasmid replication. These findings suggest that PycA indirectly regulates plasmid-mediated antimicrobial resistance by maintaining metabolic homeostasis, highlighting the link between core metabolism and extrachromosomal resistance mechanisms.

#### 3.4.3. Deletion of *pycA* Downregulates the Expression of Cell Adhesion-Associated Proteins

In the Δ*pycA* strain, the expression of multiple cell surface-associated virulence proteins was significantly reduced, including FnbA, FnbB, SasG, Fib, SpA, and IsaAB (Table 3). FnbA and FnbB mediate adhesion by interacting with host fibronectin through their C-terminal domains [49] and also promote interbacterial aggregation and biofilm formation [50]. SasG facilitates adherence via homophilic interactions and binding to glycosylated receptors on keratinocytes, promoting skin colonization [51,52]. Fib binds host fibrinogen, mediating bacterial attachment to wound surfaces and plasma proteins, and contributes to biofilm structural stability [53]. SpA binds the Fc region of IgG with high affinity, effectively blocking immune recognition and phagocytosis to facilitate immune evasion [54]. Additionally, SpA can bind to host cell surface proteins, assisting bacterial adherence and invasion [55]. The downregulation of these proteins in the Δ*pycA* strain likely impairs epithelial adhesion, biofilm formation, and immune evasion, collectively contributing to reduced virulence.

**Figure 4:**
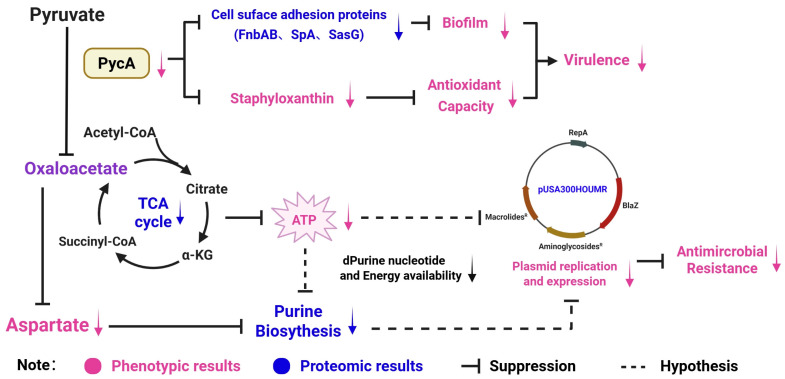
Proposed regulatory role of PycA in coordinating metabolism, virulence, and antimicrobial resistance in *S. aureus*. Purple words represent phenotypic results; blue words represent proteomic results; black solid bars (⊥) indicate suppression; and black dashed lines indicate hypothesized relationships based on indirect evidence.

Based on integrated phenotypic observations and proteomic data, we propose a hypothetical model wherein PycA functions as a central metabolic regulator, promoting both virulence and antimicrobial resistance in *S. aureus* (Figure 4). Reduced PycA activity leads to the downregulation of cell surface adhesion proteins (e.g., FnbAB, SpA, SasG) and STX biosynthesis, thereby impairing biofilm formation and antioxidant capacity—key determinants of bacterial virulence. Additionally, diminished PycA function decreases oxaloacetate production, reducing TCA cycle activity and disrupting aspartate biosynthesis. These metabolic impairments likely constrain ATP generation and purine nucleotide synthesis, leading to compromised DNA replication capacity. In response to this energy-deprived state, *S. aureus* may downregulate the replication and expression of resistance-related plasmids such as pUSA300HOUMR. This cascade ultimately results in increased susceptibility to multiple classes of antimicrobials. Collectively, these findings suggest that PycA-mediated metabolic flux is a critical upstream driver of both virulence expression and plasmid-based antimicrobial resistance in foodborne *S. aureus*.

### 3.5. Outlook: PycA as a Target for Food Safety Interventions

*S. aureus* remains a major concern in the food processing industry due to its ability to form biofilms, tolerate environmental stresses, and produce enterotoxins associated with foodborne illnesses. Disruption of *pycA* has been shown to impair stress tolerance and suppress virulence-associated traits, such as biofilm formation and STX production. These changes may increase the bacterial susceptibility to environmental stressors, including food-grade preservatives. Natural compounds, such as phenolic acids and flavonoids, have demonstrated potential to interfere with bacterial metabolism and virulence regulation [56,57,58]. High-throughput virtual screening [59] and fluorescence-based enzyme assays [60] offer a promising approach for identifying PycA inhibitors from natural product libraries. Recent studies have identified potent small-molecule inhibitors of PycA, such as 2-hydroxy-3-(quinoline-2-yl)propenoic acid, which selectively target the carboxyltransferase domain with micromolar potency [61]. In addition, biotinyl-acylsulfamide adenosine, a biotin protein ligase inhibitor, has been shown to indirectly suppress PycA activity by blocking the biotinylation of biotin-dependent enzymes [62]. While the regulatory impact of these inhibitors on *S. aureus* has not been fully defined, their potential to diminish virulence and compromise preservative tolerance suggests a novel approach to mitigating contamination in food environments. When used alongside reduced concentrations of oxidative preservatives, PycA inhibitors could enhance antimicrobial efficacy through synergistic interactions, serving as effective adjuncts to conventional preservation approaches. Furthermore, incorporating PycA-targeting agents into antimicrobial coatings, surface rinses, or packaging materials may help reduce *S. aureus* biofilm formation and persistence within food processing environments. Similar effects have been documented in previous studies on plant essential oils [63,64].

While this study provides comprehensive in vitro phenotypic evidence for the regulatory role of *pycA* in coordinating antimicrobial resistance and virulence in *S. aureus*, several limitations remain. First, our findings are based primarily on in vitro assays and a single clinical strain (BAA1717). This study may not fully capture the strain-to-strain variability or the complexity of host–pathogen interactions in vivo. Second, although consistent phenotypic and proteomic changes were observed following *pycA* deletion, the precise regulatory mechanisms by which PycA influences specific virulence pathways remain unclear. Likewise, the mechanism underlying the observed reduction in plasmid copy number of resistance gene-bearing plasmids upon *pycA* deletion lacks sufficient experimental validation. From an application perspective, translating PycA inhibitions into industrial settings poses challenges, including ensuring compound stability during food processing and achieving regulatory compliance.

Future studies should focus on identifying food-grade inhibitors and evaluating their efficacy in real food systems. In addition, in vivo studies using appropriate infection models will be essential to better understand the physiological role of PycA. Furthermore, clarifying the downstream regulatory pathways linked to PycA, particularly its interaction with resistance plasmids and virulence factors, is essential for a comprehensive understanding of its functional role.

## 4. Conclusions

This study identifies PycA as a central metabolic hub coordinating *S. aureus* virulence and antimicrobial resistance—traits critical for its persistence in food systems. By disrupting PycA, we observed attenuated biofilm formation, reduced antioxidant capacity, diminished cytotoxicity toward mammalian cells, and heightened susceptibility to antimicrobials. These findings provide a foundation for developing PycA-targeted strategies, such as metabolic inhibitors or nutrient-limiting packaging, to mitigate *S. aureus* contamination in food production and storage.

## Figures and Tables

**Table 1:** Strains and plasmids used in this study.

Name	Description	Source or Reference
Strains		
*E. coli* JTU006	Based on the *E. coli* DH10B lineage and engineered to be restriction deficient.	Han et al. [13]
*S. aureus* ATCC BAA1717	A standard MRSA strain originating from the USA300 lineage.	Teng et al. [14]
Δ*pycA*	BAA1717 mutant strain with *pycA* deletion.	This study
c*pycA*	Δ*pycA* complemented with pCL55-*pycA*, cm^R^.	This study
WT-p*pycA*	BAA1717 overexpressing *pycA* with pCL55-*pycA*, cm^R^.	This study
Plasmids		
pKZ2	Thermosensitive *E. coli*–*S. aureus* shuttle plasmid for gene deletion; Amp^R^/*E. coli*, Cm^R^/*S. aureus*.	Li et al. [15]
pKZ2-*pycA*	pKZ2-*pycA* homologous arm sequence.	This study
pCL55	Anhydrotetracycline-inducible *E. coli*–*S. aureus* shuttle plasmid; Amp^R^/*E. coli*, Cm^R^/*S. aureus*.	Karinou et al. [16]
pCL55-*pycA*	pCL55-*pycA* CDS	This study

Note: Amp^R^ indicates ampicillin resistance, and Cm^R^ indicates chloramphenicol resistance.

**Table 2:** MICs of antimicrobials against *S. aureus* strains.

Class of Antimicrobials	Antimicrobial	MIC (μg/mL)				Foldchange	
		WT	Δ*pycA*	c*pycA*	WT-p*pycA*	WT/Δ*pycA*	WT-p*pycA*/WT
β-lactams	Oxacillin	16	0.25	32	64	64	4
	Amoxicillin	4096	0.5	32	8192	8192	2
	Ampicillin	1024	1	32	2048	1024	2
	Carbenicillin disodium	1024	1	128	4096	1024	4
	Penicillin potassium	2048	4	16	4096	512	2
	Imipenem	1	0.25	4	8	4	8
	Meropenem	4	1	4	8	4	2
	Cefoxitin	64	64	64	64	1	1
Cephalosporins	Cefotaxime	16	4	32	128	4	8
	Cephalothin sodium	32	0.5	16	32	64	1
Aminoglycosides	Kanamycin sulfate	4096	2	8	8192	2048	2
	Amikacin	16	0.25	16	64	64	4
Macrolides	Erythromycin	32	0.25	1	64	128	2
	Azithromycin	64	0.5	1	128	128	2

**Table 3:** Significantly expressed differential proteins of WT vs. Δ*pycA*.

Function	Protein	Description	Accession No.	Foldchange	*p*-Value
TCA cycle	FumC	Fumarate hydratase class II	Q5HES4	0.605	0.0225
	SucC	Succinyl-CoA synthetase subunit beta	A5ISD0	0.587	0.0003
	SucD	Succinyl-CoA synthetase subunit alpha	P66866	0.565	0.0001
	Mqo	Malate quinone oxidoreductase	Q5HDJ0	0.623	0.0251
Purine metabolism	PurC	Phosphoribosylaminoimidazole-succinocarboxamide synthase	A5IRV3	0.178	0.0140
	PurM	Phosphoribosylformylglycinamidine cyclo-ligase	A5IRV8	0.300	0.0040
	PurL	Phosphoribosylformylglycinamidine synthase	A6QFS7	0.223	0.0160
	PurQ	Phosphoribosylaminoimidazole carboxylase catalytic subunit	P65904	0.225	0.0060
	PurH	IMP cyclohydrolase	Q2FI05	0.299	0.0080
	PurD	Phosphoribosylamine-glycine ligase	Q5HH10	0.305	0.0020
	PurN	Phosphoribosylglycinamide formyltransferase	Q5HH12	0.227	0.0060
	PurF	Amidophosphoribosyltransferase	Q5HH14	0.240	0.0006
	PurK	Phosphoribosylaminoimidazole carboxylase non-catalytic subunit	Q5HH19	0.252	0.0090
	PurS	Phosphoribosylformylglycinamidine synthase subunit	A0A0E1VKY1	0.248	0.0060
	PurE	Phosphoribosylaminoimidazole carboxylase	A0A5F0HIJ8	0.472	0.0820
Antimicrobial resistance	BlaZ	Beta-lactamase	D2J684	0.028	9.67 × 10^−6^
	BlaI	Beta-lactamase repressor	P0A042	0.065	0.0005
	BlaR1	Beta-lactamase sensor–transducer protein	P18357	0.001	0.0069
Cell adhesion	FnbA	Fibronectin-binding protein A	P14738	0.491	0.0105
	FnbB	Fibronectin-binding protein B	A0A0H2XKG3	0.397	0.0125
	Fib	Fibrinogen-binding protein	A6QG59	0.471	0.0006
	SasG	Surface-anchored protein G	Q2G2B2	0.418	0.0049
	SpA	Immunoglobulin G-binding protein A	Q70AB9	0.153	0.0029
	IsaA	Immunodominant staphylococcal antigen A	A6QK59	0.596	0.0001
	IsaB	Immunodominant staphylococcal antigen B	Q2FDM1	0.477	0.0019

## Data Availability

The data in the study are available from the corresponding author on reasonable request.

## Supplementary Materials

The following supporting information can be downloaded at https://www.mdpi.com/article/10.3390/foods14152566/s1, Figure S1: Time-kill curves of antimicrobials against WT BAA1717 and Δ*pycA*; Figure S2: Detailed information of the WT BAA1717 strains pUSA300HOUMR plasmid; Table S1: Minimal nutritional medium (MEM) for *S. aureus*; Table S2: Primer information used in this study.

## Abbreviations

The following abbreviations are used in this manuscript:

PycA	Pyruvate Carboxylase
MRSA	Methicillin-resistant *Staphylococcus aureus*
TCA	Tricarboxylic Acid
TSB	Tryptic Soy Broth
BHI	Brain Heart Infusion
PBS	Phosphate-Buffered Saline
STX	Staphyloxanthin
MIC	Minimum Inhibitory Concentration
CLSI	Clinical and Laboratory Standards Institute
DPPH	2,2-Diphenyl-1-Picrylhydrazyl
MEM	Minimum Essential Medium
OD	Optical Density
DMEM	Dulbecco’s Modified Eagle Medium
FBS	Fetal Bovine Serum
LDH	Lactate Dehydrogenase
SEM	Scanning Electron Microscopy
TEM	Transmission Electron Microscopy
MOI	Multiplicity of Infection
PCA	Principal Component Analysis
BCA	Bicinchoninic Acid
iRT	Indexed Retention Time
nano-UPLC	Nano-scale Ultra Performance Liquid Chromatography
DIA	Data-Independent Acquisition
FDR	False Discovery Rate
GO	Gene Ontology
KEGG	Kyoto Encyclopedia of Genes and Genomes
ppGpp	Guanosine Tetraphosphate
