# Supplementary material for: Metabolic Master Switch: Pyruvate Carboxylase Fuels Antimicrobial Resistance and Virulence in Foodborne Staphylococcus aureus
Source: Foods. 2025 Jul 22;14(15):2566. doi: 10.3390/foods14152566 (PMC12346120; doi:10.3390/foods14152566)
Supplement: Supplementary file 1 [file foods-14-02566-s001.zip › foods-3743349-supplementary.pdf]

Supplementary material

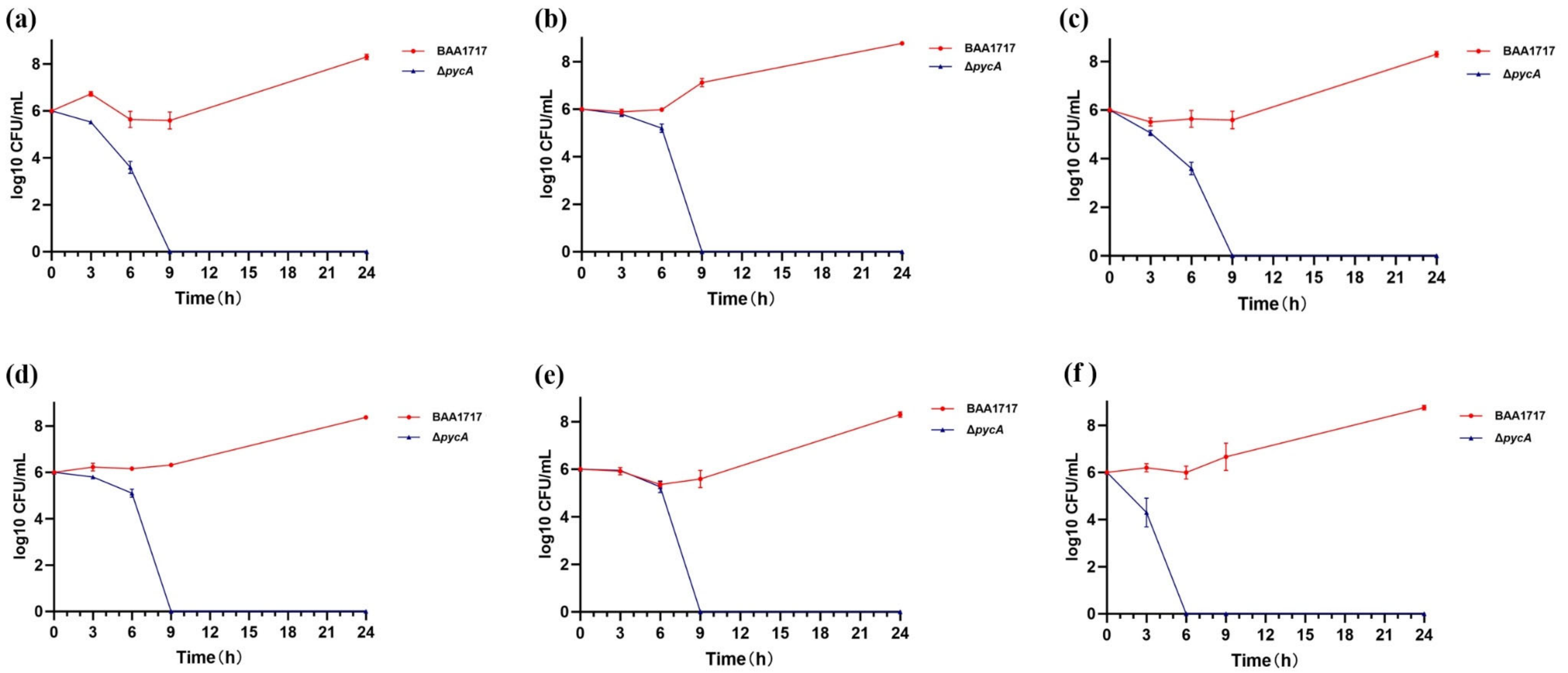

Figure S1. Time-kill curves of antimicrobials against WT BAA1717 and Δ*pycA*. (**a**)1024 μg/mL Amoxicillin; (**b**) 256 μg/mL Ampicillin; (**c**) 512 μg/mL Penicillin; (**d**) 8 μg/mL Erythromycin; (**e**) 16 μg/mL Azithromycin; (**f**) 4 μg/mL Amikacin. Antimicrobial concentrations represent 1/4 MIC values, based on the MICs determined for the WT BAA1717 strain.

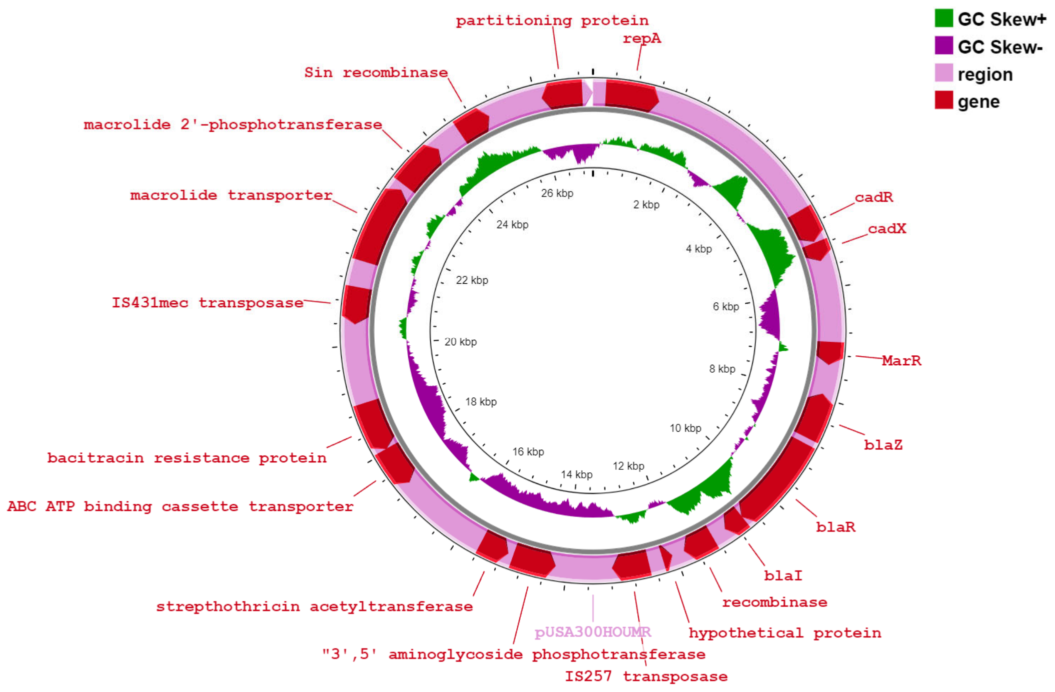

Figure S2. Detailed information of the WT BAA1717 strains pUSA300HOUMR plasmid. The plasmid carries multiple antimicrobial resistance genes, including *blaZ*, *blaR*, and *blaI* (β-lactam resistance), macrolide 2′-phosphotransferase and macrolide transporter (macrolide resistance), ABC ATP-binding cassette transporter (general efflux), streptothricin acetyltransferase (streptothricin resistance), 3′,5′-aminoglycoside phosphotransferase (aminoglycoside resistance), and a bacitracin resistance protein.

Table S1. Minimal nutritional medium (MEM) for *S. aureus*

Ingredients	Amounts per 1000 mL
**Salts:**	
KCl	3.0 g
NaCl	9.5 g
MgSO4·7H2O	1.3 g
CaCl2·2H2O	22 mg
KH2PO4	140 mg
FeSO4·2H2O	6 mg
MnSO4·4H2O	10 mg
Citric acid	6 mg
Tris Base	12.1 g
**Carbon source:**	
Glucose	5 g
**Amino acids:**	
L-Arg	125 mg
L-Pro	200 mg
L-Glu	250 mg
L-Val	150 mg
L-Thr	150 mg
L-Phe	150 mg
L-Leu	150 mg
L-Cys	80 mg
**Vitamins:**	
Biotin	0.1 mg
Thiamin	2 mg
Nicotinic acid	2 mg
Calcium pantohenate	2 mg

Table S2. Primer information used in this study

Primer name	Sequence 5′ to 3′	Purpose
RT-16s-F	GCTGCCCTTTGTATTGTC	RTqPCR
RT-16s-R	AGATGTTGGGTTAAGTCCC	
RT-*repA*-F	GCTGGGTTGATGAAGATGGT	RTqPCR/qPCR - *repA*
RT-*repA*-R	CCTTCTCGCCGTAAGGTTCA	
RT-*blaZ*-F	AGGTTCAGATTGGCCCTTAGGA	RTqPCR/qPCR-*blaZ*
RT-*blaZ*-R	GCTGCTTTCGGCAAGACTTT	
RT-*pycA*-F	TGAACAATTTGCGCGTCGTT	RTqPCR/qPCR - *pycA*
RT-*pycA*-R	TGGACCGTCTGTACCAGGAA	
*kpn*I-*pycA*-F	CGGGGTACCTCATTGCTTGCGATAGGTAAC	pkz2-Δ*pycA*
*SaL*I-*pycA*-R	ACGCGTCGACAATCGCCTGTCGCTATTGTG	
pycA-up-R	TTCTAAGGTGGCGTGACTGTGCTGATGTCTAATTCTG	
pycA-down-F	AGACATCAGCACAGTCACGCCACCTTAGAACTGATAT	
T*pycA*-F	AACGGAAGTTAGGCAACCAA	
T*pycA*-R	GAATACTGTCCACCAGGCATT	
c*pycA*-F	AATTAAAATAAGCTTGATAGGAGGACAGCTTTGAAACA AATAAAAAAGTTACTTG	pCL55-c*pycA*
c*pycA*-R	CGTCTTCAAGAATTCGATTTAGTCAGTTGCTTTTTCAATT TC	
pCL55-F	ACATCATTAATTCCTCCTTTTT	
pCL55-R	TTTATCAGGGTTATTGTCTCAT	
